# Gene expression profiling of *brakeless* mutant *Drosophila* embryos

**DOI:** 10.1016/j.dib.2015.08.033

**Published:** 2015-09-05

**Authors:** Filip Crona, Bhumica Singla, Mattias Mannervik

**Affiliations:** Department of Molecular Biosciences, The Wenner-Gren Institute, Stockholm University, Stockholm, Sweden

**Keywords:** Transcription, Transcriptional co-regulator, *Drosophila* embryo, Brakeless, Scribbler, Master of thickveins

## Abstract

The transcriptional co-regulator Brakeless performs many important functions during *Drosophila* development, but few target genes have been identified. Here we use Affymetrix microarrays to identify Brakeless-regulated genes in 2–4 h old *Drosophila* embryos. Robust multi-array analysis (RMA) and statistical tests revealed 240 genes that changed their expression more than 1.5 fold. We find that up- and down-regulated genes fall into distinct gene ontology categories. In our associated study [2] we demonstrate that both up- and down-regulated genes can be direct Brakeless targets. Our results indicate that the co-repressor and co-activator activities of Brakeless may result in distinct biological responses. The microarray data complies with MIAME guidelines and is deposited in GEO under accession number GSE60048.

**Specifications Table** *[please fill in right-hand column of the table below]*

**Table:** 

Subject area	Biology
More specific subject area	Developmental Biology, Molecular Biology
Type of data	Table and figure
How data was acquired	Affymetrix *Drosophila* gene chip (version 2) array
Data format	Analyzed with RMA and statistical tests
Experimental factors	Comparison of control embryos and embryos derived from brakeless germline clones
Experimental features	RNA was extracted from 2–4 h old embryos, converted to cDNA and hybridized to Affymetrix arrays
Data source location	
Data accessibility	**The data is deposited in GEO under accession number GSE60048.**
http://www.ncbi.nlm.nih.gov/geo/query/acc.cgi?token=olqdikeqjnutraf&acc=GSE60048

**Value of the data**

• This data significantly extends the number of Brakeless-target genes
• The data shows that more genes are down-regulated than up-regulated in *brakeless* mutant embryos
• Down-regulated and up-regulated Brakeless target genes fall into distinct gene ontology clusters

## 1 Data

**Fig. 1:**
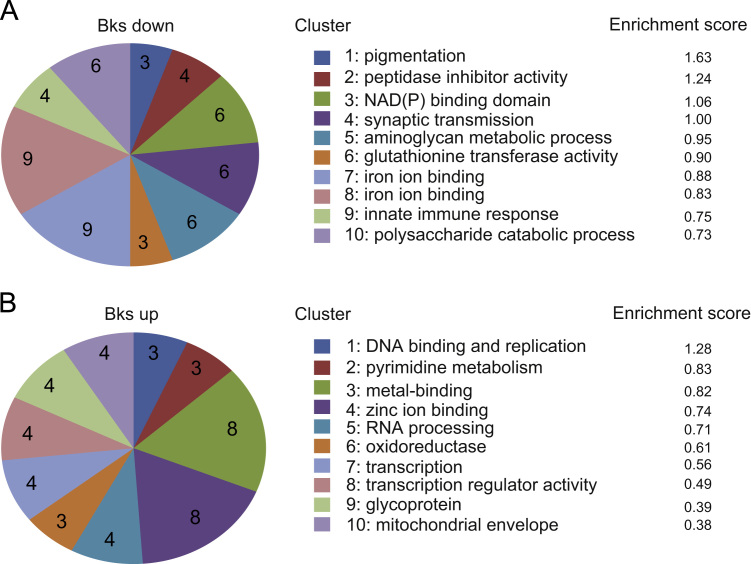
Brakeless-repressed and Brakeless-activated genes fall into distinct GO-categories**.** Grouping of genes into enrichment clusters revealed by DAVID functional annotation analysis. (A) Genes down-regulated in *bks* mutant embryos from the expression array and (B) up-regulated genes. The enrichment score depicts the geometric mean in log_2_-scale of the member's *P*-values within a cluster.

To identify Brakeless target genes in the early embryo, RNA was isolated from embryos derived from *brakeless* (*bks*^*278*^) germline clones, which lack the maternal contribution of Brakeless. This was compared to RNA from germline clone embryos generated with the unmutagenized FRT chromosome on which the *bks*^*278*^ allele was induced [8]. The RNA was converted to cDNA and hybridized to an Affymetrix array. Mis-regulated genes that change their expression more than 1.5 fold were identified (supplementary material Table 1). We compared our gene list to an RNA-seq dataset that distinguishes maternal from zygotic transcripts in *Drosophila* embryos using polymorphisms [7]. The Brakeless-regulated genes were categorized as being maternally, zygotically, or maternally and zygotically (matzyg) derived (supplementary material Table 1). They were also subjected to functional annotation analysis using DAVID [3], which groups genes into clusters based on co-association with gene ontology (GO) terms (supplementary material Table 1). As shown in Fig. 1, up-regulated and down-regulated gene fall into distinct GO clusters.

## 2 Experimental design, materials and methods

### 2.1 Germline clones

The FLP–FRT dominant female sterile technique previously described in Ref. [1] was used to generate *brakeless* germline clones. The *bks*^278^ allele was used, which has a 345 bp deletion that causes a frame shift at aa 741 resulting in addition of 79 novel amino acids [5]. It was outcrossed with a *w*^*1118*^ strain to remove potential second-site mutations. *FRT*^*2R*^*G13 bks*^*278*^*/CyO* females were crossed with males of the genotype *hs-FLP/Y; FRT*^*2R*^*G13 ovo*^*D1*^*/CyO,* derived from *FRT*^*2R*^*G13 ovo*^*D1*^*/T(1;2)OR64/CyO* (Bloomington stock #4344). Offspring larvae were heat-shocked for 3 h at 37 °C on days 3, 4 and 5 after egg-laying to induce expression of the FLP recombinase. *Cy+* females were crossed to *FRT*^*2R*^*G13 bks*^*278*^/*CyO* males and embryos were collected, dechorionated using bleach, and directly frozen in Trizol at −80°C for RNA isolation. Corresponding crosses, embryo collection and RNA isolation was performed with an unmutagenized *FRT*^*2R*^*G13* chromosome.

### 2.2 Microarray analysis

Staged 2–4 h old embryos were collected and immediately frozen at −80°C prior to RNA extraction. Total RNA was isolated using TRIzol (Invitrogen) and purified using an RNeasy kit (Qiagen) according to the manufacturer's protocols. Forty ul of embryos were used for each of three biological replicates of embryos derived from *FRT*^*2R*^*G13 bks*^*27*8^ or *FRT*^*2R*^*G13 c px sp* control germline clones. cDNA probes were hybridized to an Affymetrix *Drosophila* gene chip (version 2). The intensity values were normalized and summarized with the robust multi-array analysis (RMA) method [6], using R (www.R-project.org) and the Bioconductor package [4].

### 2.3 Statistical analysis

After RMA normalization, 640 probes passed *a*>1.5 fold difference in median expression levels between the conditions investigated. They were subjected to a two tailed unpaired Student´s *t*-test with a 95% confidence level, followed by Bonferroni´s correction for multiple tests, resulting in a *P*-value cut-off at 0.000078125. After removal of probe-sets targeting duplicates and pseudogenes, 240 genes remained.

### 2.4 GO analysis

The lists of genes with significantly changed expression levels containing 174 down- and 66 up-regulated identifiers were used as input lists for the DAVID Functional Annotation Clustering tool [3]. The tool provides analysis of annotation content and gene ontology term enrichments, to highlight the most relevant GO terms associated with a gene list. The enrichment score is a geometric mean of the member´s *P*-values in a -log scale within an annotation cluster.

## Appendix A Supplementary material

Supplementary material Table 1. **Brakeless*****-*****regulated genes in 2–4 h old embryos.** Gene expression changes between embryos derived from germline clones with a *FRT*^*2R*^*G13 bks*^*278*^ chromosome or a *FRT*^*2R*^*G13* control chromosome as determined by an Affymetrix microarray. Only genes with a statistically significant fold change in expression levels (fold change>1.5, *P*<0.05) are included in the list. Column D displays fold change in expression, and column E contains the *P*-values after Student’s t-test with Bonferroni multiple correction (95% confidence interval, *P*<0.05/640). In column F the RMA normalized log_2_ expression levels for *FRT*^*2R*^*G13* wild-type are shown. Column G shows classification of transcripts into zygotic, maternal or matzyg according to polymorphisms [7]. Gene Ontology information in columns H and I originate from FlyBase. Column J contains classification of genes into enrichment clusters (see Fig. 1) using the DAVID functional annotation tool based on annotated association with Gene Ontology terms.
